# Atomic Force Microscope Cantilever Flexural Stiffness Calibration: Toward a Standard Traceable Method

**DOI:** 10.6028/jres.116.015

**Published:** 2011-08-01

**Authors:** Richard S. Gates, Mark G. Reitsma, John A. Kramar, Jon R. Pratt

**Affiliations:** National Institute of Standards and Technology, Gaithersburg, MD 20899

**Keywords:** AFM, calibration, cantilever, spring constant, stiffness

## Abstract

The evolution of the atomic force microscope into a useful tool for measuring mechanical properties of surfaces at the nanoscale has spurred the need for more precise and accurate methods for calibrating the spring constants of test cantilevers. Groups within international standards organizations such as the International Organization for Standardization and the Versailles Project on Advanced Materials and Standards (VAMAS) are conducting studies to determine which methods are best suited for these calibrations and to try to improve the reproducibility and accuracy of these measurements among different laboratories. This paper expands on a recent mini round robin within VAMAS Technical Working Area 29 to measure the spring constant of a single batch of triangular silicon nitride cantilevers sent to three international collaborators. Calibration techniques included reference cantilever, added mass, and two forms of thermal methods. Results are compared to measurements traceable to the International System of Units provided by an electrostatic force balance. A series of guidelines are also discussed for procedures that can improve the running of round robins in atomic force microscopy.

## 1. Introduction

Atomic force microscopy (AFM) has been struggling with issues of calibration and accuracy since its inception in 1986 [1]. Initially, the emphasis was on dimensional accuracy since imaging was the first focus of AFM. The nonlinearity inherent in the piezoelectric ceramics used to drive most AFM scanners was addressed by either software corrections or incorporation of closed loop sensors. More recently, interest in using AFM to measure nano-scale forces has prompted researchers to deal with AFM cantilever spring constant calibration issues. This has also led manufacturers to develop better cantilevers with tighter production tolerances (and hence a smaller spread in spring constants) and researchers to develop better methods to calibrate cantilevers in the field.

As spring constant calibration techniques have proliferated, attempts have been made to compare techniques to determine which ones have the best precision and accuracy. Efforts have also begun in standards organizations such as the Versailles project on Advanced Materials and Standards (VAMAS), and the International Organization for Standardization (ISO) to understand which techniques are useful for comparing data from different laboratories around the world. One of the Authors (RG) is currently Chairman of VAMAS Technical Working Area 29 on Nanomechanics Applied to Scanning Probe Microscopy. Recently a mini round robin (MRR) was conducted among three national laboratories worldwide in an attempt to provide a foundation for a larger round robin on comparison of flexural stiffness calibration methods for AFM. This MRR had several key findings that were useful in streamlining a larger round robin (currently underway) and minimizing the possibility of damage to the samples during shipping and handling. A summary report is available online [2].

The purpose of this paper is to expand on the details of the experimental measurements that were made during the MRR and also provide additional data on the same cantilevers using techniques that were not available at the time of the MRR in order to establish the potential accuracy of the techniques.

## 2. VAMAS TWA 29 Mini Round Robin

The MRR was conducted among three laboratories in three countries to evaluate handling and testing protocols for determining the flexural spring constants of AFM cantilevers. The laboratories were National Laboratories in the United States (The National Institute of Standards and Technology—NIST), the United Kingdom (The National Physical Laboratory—NPL), and Japan (The National Institute for Material Science—NIMS) and included researchers who were very familiar with AFM. The study was intended as an initial foray into cantilever calibration in order to experience logistical, handling, and testing issues that might come up in a larger round robin with many different participants. By experiencing and addressing problems in the MRR it was anticipated that a future round robin could be conducted with fewer problems.

A kit consisting of six similar commercial test cantilevers from a single production batch (silicon nitride, triangular) and a commercial reference cantilever artifact, was mailed to each laboratory in sequence. A detailed description of these cantilevers is provided in Appendix A. Each laboratory was asked to perform cantilever spring constant calibration procedures on the test cantilevers using procedures with which they were familiar. Drafts of very detailed procedures for an added mass method and a reference cantilever method were written by two of the authors (RG & MR) and were included with the test kit and are also attached to this paper as Appendices B and C. These procedures included explicit instruction on how to calculate the spring constant in each case and report the values. The results of the MRR were collected and the data compared.

All three laboratories performed the reference cantilever method and the statistical analysis of the results of each laboratory indicated good agreement between the results obtained from the three laboratories. One of the laboratories (NIST) also conducted added mass calibrations, and the spring constant values obtained were consistent with those obtained with the reference cantilever method. These results are described in more detail below.

## 3. Reference Cantilever Method

The reference cantilever method is a straightforward method for obtaining a spring constant for an unknown, test, cantilever by performing a force curve on a known spring. By measuring the deflection of the known—unknown cantilever couple, the spring constant of the unknown can be calculated. This technique was originally popularized by Tortonese & Kirk [3] who produced some of the first microfabricated reference cantilevers used specifically for this purpose. In practice in an AFM, the technique actually requires two force curves. One on an extremely stiff surface approximated as rigid essentially the z piezo displacement effect on the laser spot translation across the photodiode (the so-called optical lever sensitivity) while the other force curve on the end of the reference spring (the reference cantilever) provides the relationship between displacement and laser spot translation for the springs in series. The defining equation used to estimate the spring constant of the unknown (test) cantilever is:

(1) $$\begin{array}{l} \;k_{\text{test}}=k_{\text{ref}}\left(\frac{S_{\text{rigid}}}{S_{\text{cant}}}-1\right)\cos^{2}\varphi \\ \text{where}\;k_{\text{ref}}=k_{\text{end}}{\left(\frac{L}{L-\Delta L_{\text{tip}}}\right)}^{3}. \end{array}$$

*S*_rigid_ and *S*_cant_ are the slopes of the compliance curves during contact with either the “rigid” (a very stiff piece of Si) or reference cantilever surfaces and typically have units of V/nm. The cosine squared correction is needed to correct geometrically for the inclined angle (*φ*) of the test cantilever in the AFM holder. In the case of an 11° incline, this works out to be about a 4 % correction. Note that for cantilevers that have very long tips relative to their lengths the geometric correction becomes more complex and the torque of the tip must also be taken into account [4]. For the case of the MRR, the test cantilevers used have short tips (3 μm) and they are relatively long (115 μm) so this effect is at the subpercent level and can be ignored. The second half of equation 1 represents the “off end correction” factor that must be applied. Spring constants for reference cantilevers are usually specified at the very end of the cantilever and it is not possible to actually contact there. By pressing at a specific and known location, the actual stiffness at that location can be used based on the Euler-Bernoulli model for a rectangular cross section cantilever which varies as the cube of the length.

The spring constant calculated in the reference cantilever method above is the “intrinsic” spring constant—i.e., perpendicular to the long axis of the test cantilever. As such, it is portable and can be used in any AFM instrument by just dividing by the cos^2^ *φ* of the inclined angle to give the vertical (“effective”) component of the spring constant for that particular instrument.

One key aspect of the reference cantilever procedure is the care needed in defining the precise point of contact along the reference cantilever. This feature was considered essential because the spring constant for a rectangular reference cantilever varies as the cube of the length. Small errors in placement of the contact point can therefore have large effects in measured stiffness (precision and accuracy). One approach, used successfully over the years in our laboratory uses a known length (the tip set back length, Δ*L*_tip_) as a visual internal standard to position the contact point. This alignment procedure, provided in Appendix B, was also provided to the MRR participants. The reference cantilever procedure provided in Appendix B did not place any constraints on which force curve (approach or retract) was to be used for the slope estimation. It was suggested that force curve ramp length start at 500 nm, but it could be adjusted to suit the requirements of the particular instrument. A minimum of six measurement pairs (on a “rigid” surface and on the reference cantilever) were requested from each participant to determine the statistical repeatability of the measurement from each laboratory^1^.

The main drawback in the reference cantilever method is that it requires the AFM tip to actually touch the surface during use. This can potentially cause damage to the tip that may affect future use. Researchers often get around this by performing the calibration *after* the more delicate imaging and measurements have been conducted. A second caution is that the reference cantilever calibration value is only as accurate as the reference cantilever itself. Variations in reference cantilevers as great as 30 % have been observed [5, 6] so care must be taken to ensure that accurate values are used. One significant advantage of the reference cantilever method is that it has the potential to be traceable to the International System of Units (Système International d’Unités or SI). Work is also currently underway at NIST to microfabricate a large batch of reference cantilevers (NIST SRM 3461) that would be very uniform and statistically linked to (SI) traceable measurements to reduce the current accuracy uncertainty in reference cantilevers.

## 4. Initial VAMAS Data Comparison

**Fig. 1:**
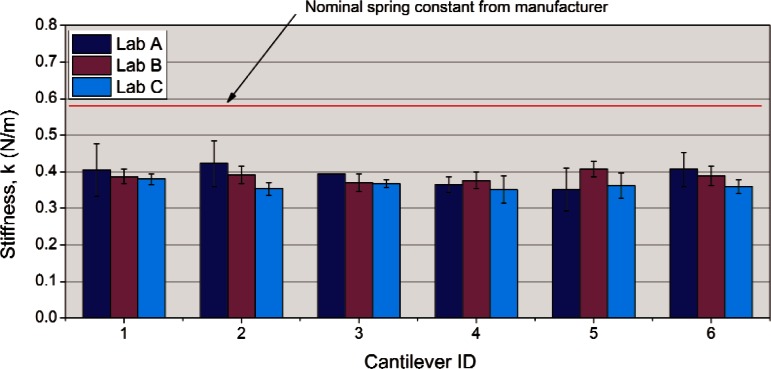
Comparison of reference cantilever calibration results from three different laboratories.

As all three labs utilized the reference cantilever method, the collated data can be easily compared and are summarized in Fig. 1. The error bars in each sample represent the standard deviation of the mean using the six repeat data on each specific cantilever. The three labs used different AFM instrumentation. Lab “A” used a Veeco^2^ Dimension 3100, lab “B” used a Veeco Multimode, and lab “C” used a Park XE100 system.

The average relative uncertainty of the measurements for all six cantilevers was 11 % (for lab “A”) and 6 % (for labs “B” and “C”). Three things are immediately apparent from the graph. First, all three labs had statistically similar results and therefore the reproducibility of the prescribed method from lab to lab was very good. Second, the six test cantilevers were similar in spring constant suggesting that for these particular cantilevers selecting a subset of cantilevers within a single manufacturing batch (based on resonance frequency for example) can be fairly effective at reducing the cantilever-to-cantilever variation. Third, the spring constants estimated from the reference cantilever method were all lower than the nominal value assigned by the manufacturer by about 30%.

While the average repeatability of the measurements for lab “A” was typical for this type of measurement reported in the literature [7] (± 10 % to ± 30 %), the observation of the variation of error bars for lab “A” suggests a discreteness in the data that points to a statistical analysis issue. Test #1 had a relative uncertainty of ± 17 % while test #3 had no uncertainty. Looking at the raw data indicated that the problem lay with the low number of reported significant digits (two) from that laboratory. Since the actual numbers reported for the slope of the force curve measurement in Eq. (1) (e.g. 0.014 V/nm) had a small first digit, change of just one digit in the second number is a change of almost 10 %. The effect is to blow up or nullify small variations in the data, depending on where the data lay relative to the last significant digit and explains the discrete nature of the data. This highlights the need to specify a minimum number of significant figures (in this case three) for the raw data in the round robin.

The procedure for this MRR did not specify whether to use the approach or retract portion of the force curves to calculate the slope of the compliance curves. In retrospect, that was a dangerous omission that could have affected the comparison of data from different laboratories. It was fortunate that the selected test cantilever had a short (nominally 3 μm) tip that had retract curves that were very similar in slope to approach curves so it didn’t matter, but that will not always be the case. Pratt et al. [8] showed that in some circumstances, approach and retract slopes can be very different and the spring constants calculated from them will vary. They attributed the effect to friction between the tip and surface that gets amplified by the geometric leverage of tip height and causes hysteresis in the force curves. They recommended an average of both curve slopes be used to reduce the influence of the effect on the estimated spring constant.

The absolute value of the spring constant of the test cantilever obtained in the reference cantilever method is based on the value for the reference cantilever; therefore, the accuracy of the method depends on the accuracy of the reference cantilever. In the case of this study, that absolute value was based on the manufacturer’s nominal spring constant value which was given as “0.711 N/m” for all five “long” reference cantilevers in the set. For the purposes of comparing test results among the three laboratories, the actual absolute value did not matter since all participants used the exact same artifact (the long reference cantilever). Essentially, the comparison provides the relative precisions of the calibrations and how they might be biased by the instruments themselves.

The reference cantilever used in the MRR was selected from a batch of five cantilever sets purchased from the manufacturer (CLCF-NOBO, Veeco Probes, Camarrillo, CA) and utilized the long reference cantilever with a resonance frequency closest to the nominal value specified in the hope that the nominal spring constant would represent a more accurate value. An estimate of the spring constant of the reference cantilever, performed using the Sader method [9] using the web-based Java applet [10] indicated a stiffness of 0.70 N/m, which was close to the nominal value. This suggested that the manufacturer-assigned value for the MRR was reasonable for that particular cantilever. Another long reference cantilever from the same purchased set had a Sader-estimated [10] stiffness of 0.57 N/m so there may be significant variation from chip-to-chip even in a single manufacturer’s batch.

More recently, one of the authors (RG) has been developing the capability at NIST to run the thermal method [11] using laser Doppler velocimetry (LDV). Using this technique, the stiffness of the reference cantilever used in the MRR was measured as 0.734 N/m ± 0.006 N/m which is only 3 % greater than the nominal value originally used for the MRR. The reference cantilever method values provided in this paper assume the original 0.711 N/m values.

## 5. Added Mass Method

A second calibration method, the added mass method, was utilized at NIST (laboratory “B”) using the same commercial AFM instrument that was used for the reference method. The method was originally developed by Cleveland [12] to calibrate the spring constant of a cantilever using only the resonance frequency measurement of a series of experiments where small, known, masses are added to the end of the cantilever. The exact procedure is described in detail in Appendix C. The technique requires some skill on the part of the operator to be able to apply and remove tungsten or gold microspheres on a cantilever without damaging it but the precision of the resulting data is quite good. The largest uncertainty in the overall process lies with the estimation of the added mass which depends mostly on the measurement of the diameter of the spherical mass added. We typically use a calibrated optical microscope with digital image capture capabilities to estimate both the sphere diameter and the actual location of the sphere on the cantilever (for the offset correction explained in Appendix C). It should be noted that the spring constant estimated with the added mass method is the intrinsic one and no angle correction is needed.

One significant advantage of using the added mass method is that it does not require touching the test cantilever tip to a surface during calibration. Its major drawback is the complexity of the process and the skill required to carefully place microspheres onto the test cantilever surface and remove them without damaging the cantilever.

**Fig. 2:**
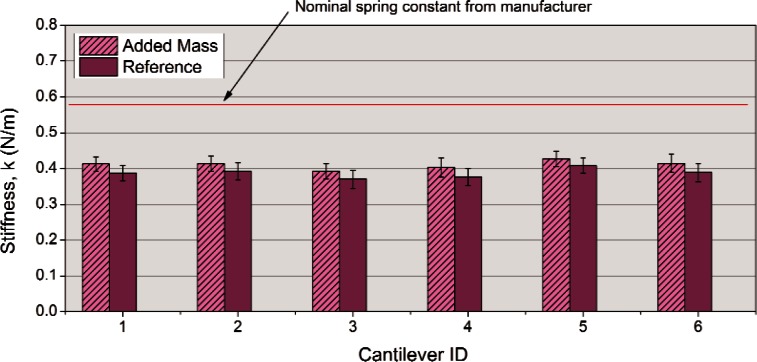
Comparison of added mass and reference cantilever methods for laboratory “B”.

The results obtained on all six test cantilevers are compared to the reference cantilever method results in Fig. 2. The relative uncertainty of the added mass method was estimated at ± 6 % and is typical for the authors’ experience with this method.

The results of the two methods agree well statistically and reinforce the previous observation that the six test cantilevers are very similar in spring constant and that the measured calibration values are about 30 % less than the nominal values reported by the manufacturer. Even though the two data sets are statistically equal, there is a consistent trend of the Reference Cantilever data being slightly less in stiffness than the added mass data for all six samples that points to a consistent bias. This can be partially explained by the use of the nominal 0.711 N/m reference cantilever calibration value used for the MRR. If the value of 0.734 N/m (obtained by LDV Thermal) is used instead, the data corrects upward by 3 % and the gap between the two data sets decreases by 50 %. While the scope of the MRR was limited to looking at the precision of the calibration methods and not the accuracy, the numerical agreement of the two methods was a positive sign that these two techniques may also be accurate.

## 6. Additional Calibration by Thermal and EFB Methods

Three additional techniques were subsequently used to estimate the spring constants of two of the VAMAS test cantilevers. One method, the thermal method, as implemented in an AFM, was performed using two different commercial AFM systems. A Veeco Multimode AFM (Veeco Instruments, Santa Barbara, CA) which was also used for the added mass and reference cantilever methods in the MRR is described as AFM1. A second commercial instrument—an Asylum MFP-3D (Asylum Research, Santa Barbara, CA) standalone is described in this paper as AFM2. The second spring constant calibration technique was an experimental version of the thermal method we have been developing at NIST that utilizes laser Doppler vibrometry—LDV (MSA500, Polytec USA, Hopkinton MA) to measure the power spectrum for the flexural resonance mode of the cantilever. The third calibration technique used was the electrostatic force balance (EFB) [13]. This instrument, designed and developed at NIST, is capable of measuring nanonewton forces applied to surfaces, and can measure spring constants with both good precision and accuracy, since it is SI traceable.

The thermal methods are all based on the original work of Hutter and Bechhoeffer [11] and later refined by several researchers [14, 15]. Based on the equipartition theorem, the thermal method is an energy balance in which the spring constant is obtained through the potential energy term. The technique relies on the measurement of the frequency spectrum obtained while the cantilever is in thermal equilibrium with its environment. Typically, these thermal vibration amplitudes are quite small, and very sensitive, high speed, electronics are required for accurate measurement of both the frequency and vibrational amplitude.

The thermal methods that were implemented on commercial AFM’s used the standard setting recommended by the instrument manufacturers which included a setting of 1.09 for the “chi” [16] correction factor for the Asylum instrument. This correction factor takes into account the effect of the optical lever detection system used in most AFM instruments which actually measure angle changes in the cantilever end and not absolute deflection. The equation used to calculate the spring constant is:

(2) $$k=\frac{0.971k_{B}T}{X^{2}\langle z_{1}^{2}\rangle },$$

where *k*B is the Boltzman constant, *T* is the absolute temperature, and the $<{z_{1}}^{2}>$ term represents the mean squared displacement of the first bending mode of the cantilever. The first (0.971) term is a mode correction factor that accounts for the first mode displacement contribution of the cantilevers [14] and is small (only 3 %) compared to the almost 20 % for the chi factor correction. Note that the mode correction factor of 0.971 used represents an ideal case for rectangular cantilevers as a simplification. Stark et al. [17] used finite element analysis to estimate the mode correction factor for a particular commercial triangular cantilever (different from the one used in this MRR study) and obtained a value that was slightly lower (0.963).

The chi factor setting used for the Veeco Multimode instrument was not stated but based on an application note [18] from the manufacturer it appears to be the same (1.09) value. AFM thermal calibration also requires a force curve be applied to an infinitely stiff surface to determine the optical lever sensitivity once the laser spot is aligned on the cantilever. This determination suffers from the same issues present in the reference cantilever method and in some cases, friction can cause hysteresis in the approach-retract curves and affect the calibration uncertainty. Once the optical lever sensitivity is obtained, precision of this method is usually quite good (repeatability of a percent or two). It should also be pointed out that because the optical lever sensitivity calibrates the vertical deflection of the tilted cantilever it estimates the vertical component of the spring constant (the “effective” spring constant). This value must be multiplied by cos^2^ *φ* (where *φ* is the inclined angle of the test cantilever) to provide the intrinsic spring constant.

The thermal method being developed on a laser Doppler velocimeter at NIST does not require touching a surface to conduct a force curve since it is already calibrated for deflection in the *z* direction. It also does not require mounting or tilting of the cantilever so actual cantilever handling can be eliminated and there is no tilt correction component to the calculation. As a result, the mode correction factor [14] is the only adjustment necessary to the spring constant calculated (about 0.97 or 3 % downward adjustment). The repeatability of the measurement varies slightly with type of cantilever but is approximately ± 2 %. The authors are currently cross checking the spring constants obtained using LDV Thermal and EFB to determine the actual accuracy of the LDV thermal method.

The EFB method [13] was developed at NIST to provide SI traceable nanonewton force measurements. The key components are an extremely sensitive electrostatic force transducer combined with an interferometer which allow simultaneous measurement of ultra-small force and displacement as a surface (a flat diamond) is pressed against the measurement feature (in this case the tip of the cantilever). Other key aspects of the instrument include isolation from noise and environmental influences. The instrument is housed in a vacuum chamber, located in a ± 0.1° C temperature controlled NIST metrology laboratory, twelve meters underground. This facility is part of the NIST Advanced Measurement Laboratory—AML. The only major drawback to the EFB method is that it requires touching the AFM tip onto a surface. Since measurements are performed in vacuum, the meniscus forces which can be substantial at these force scales are minimized.

**Fig. 3:**
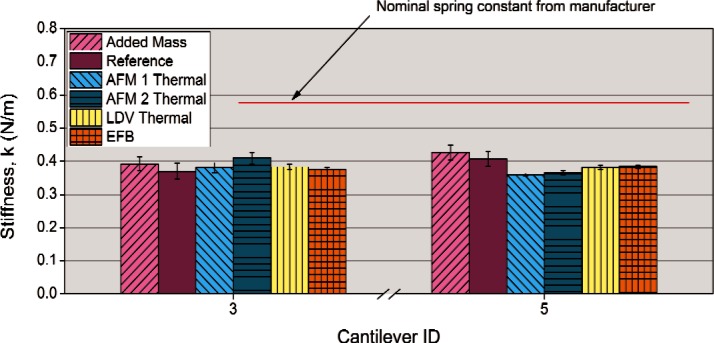
Comparison of spring constants estimated for all methods used at NIST for two test cantilevers (#3 and #5 from the VAMAS MRR).

The results of all of the spring constant measurement techniques are summarized for two VAMAS MRR test cantilevers (#3 & #5) in Fig. 3. Since the EFB is SI traceable, it provides a benchmark for the absolute accuracy for each technique for these particular cantilevers. First, it becomes apparent that the actual spring constant of each cantilever is much less than the manufacturer’s nominal value which confirms that nominal values are just estimates and may be off by large amounts. Second, it is encouraging that all of the techniques used provide reasonably similar estimates of the spring constants for each cantilever. If the uncertainties were relaxed to ± two standard deviations (95 % confidence limits) most of the results would be statistically similar. Under tighter scrutiny it appears that the results of the EFB and the LDV thermal are identical and indicate that the LDV method as applied to these cantilevers is both precise and accurate.

If we take the EFB value as the benchmark for accuracy we can tabulate the results and provide both a precision (Type A or statistical random uncertainty) and accuracy (as a deviation from the accurate benchmark EFB value and categorized as Type B or systematic uncertainty) estimate for each technique.

Overall, these results demonstrate that for these types of test cantilevers, there are several good methods that can be used to calibrate the spring constant. Each technique has its strengths and weaknesses.

The EFB was used as the accuracy benchmark because it is SI traceable. While it may be the most precise and accurate technique, measurements must be made under vacuum after thermal equilibration which can take days. Data acquisition and analysis is rigorous and time consuming such that it may take a week of experimentation and analysis to produce a single value. The tip actually contacts a surface during the measurement.

For the cantilevers tested, LDV thermal analysis seems to have a precision and accuracy, comparable to that of the EFB. This is especially useful since the LDV thermal method does not require that the tip actually touch a surface during the measurement. Given the much greater ease of use, this technique would seem a very desirable tool in a cantilever spring constant calibration method arsenal. The authors are currently collaborating on investigating the accuracy and precision of this method for a wider range of cantilever types and spring constants to see if the results observed in this study persist.

The AFM instrument Thermal techniques seem reasonably capable of determining spring constants accurately (systematic uncertainty within ± 10 %). The advantage of this technique is that it can be done in the AFM instrument either just before or just after the experimental measurement of interest. We believe that much of the variation in precision and accuracy was due to uncertainties in the optical lever sensitivity measurements, obtained when the cantilever actually touched the surface.

The reference cantilever method was fairly precise (random uncertainty of 5 % to 7 %) and also within 6 % of the accurate value using the manufacturers estimated spring constant of 0.711 N/m. If we instead use the value of 0.732 N/m measured by the LDV Thermal method performed on the reference cantilever the absolute spring constants will adjust upwards 3.2 % resulting in a measurement bias of + 1 % and + 9 % for test cantilevers #3 and #5 respectively.

The Added Mass method was also reasonably accurate (only 4-11 % difference from the benchmark) and precise (relative random uncertainty of 5 %). This method also has the advantage that it does not require the tip to contact the surface during the measurement. This is offset somewhat by the more complex nature of handling microspheres and placing them on the test cantilever.

## 7. Handling and Use Damage

The initial lesson learned from the MRR was that sharing delicate samples among participants poses dangers to the outcome of the study in several ways. First, these test chips must be physically handled in order to make a measurement in an AFM. This involves picking up the small chips with tweezers and carefully orienting them in the appropriate AFM chip holder and securing them (usually with a small spring clip). There is always the danger that participants can accidently drop the test sample which would usually break the cantilever and then all future data from that sample would be unavailable. There is also danger that the mere act of squeezing the test sample chip with the tweezers can cause fracture damage to the chip and create debris which can settle on part of the cantilever and affect the measurement in a variety of ways, from changing the resonance frequency to changing the reflectivity of the laser on the back of the cantilever. Second, damage can be introduced on use either through wear or accidental contact during alignment or calibration.

In the MRR, the reference cantilever was supplied pre-mounted on a steel puck to eliminate the need to directly handle that particular chip. Despite this precaution, inspection of the reference cantilever at NIST after all of the testing revealed that two of the three original reference cantilevers on the handle chip (ones not actually used for calibration in the MRR) had been broken off during use. One feature of AFM’s is that there is usually a limited view of the intended point of interaction and if there are other cantilevers on the same chip (especially longer cantilevers), they can be inadvertently contacting surfaces out of view. It was thought that additional cantilevers on the chip that were not tested may have inadvertently contacted the unused reference cantilevers, breaking them off.

**Fig. 4:**
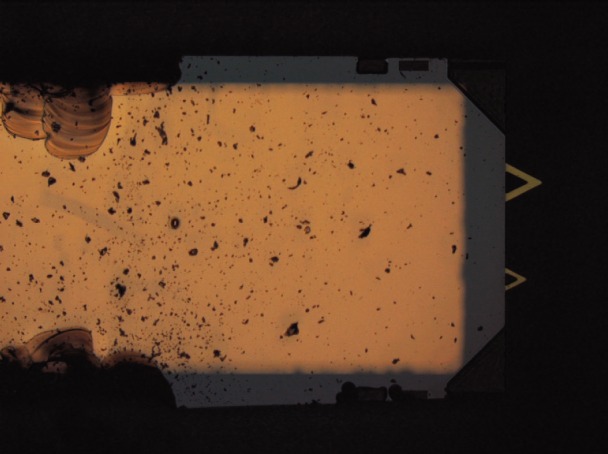
Handling damage on AFM chip used in the mini round robin.

Inspection of the test cantilevers after the MRR also revealed significant chipping damage on the edge of the chip and significant amounts of debris particles on surfaces of the chip (Fig. 4). Additional scanning electron microscopy imaging of the cantilevers themselves confirmed that the debris did indeed make its way onto the cantilever as well. As the fracture damage seemed to be caused by stress concentrations imposed by the forceps during handling of the chip some suggestions are offered to try to reduce these effects. It is suggested, for example, that future handling procedures specify a particular type of forceps with a flat paddle end that does not produce as high a stress concentration on the sides of the chip during handling as pointed forceps. In addition, a method is suggested for removing the test chips from the adhesive gel used for transportation by gently twisting, rocking, and pealing the chip off the gel. This method should reduce the forces needed to extract the chips from the storage case and will ultimately reduce the amount of debris generated during sample handling. Inspection of the tips of the test cantilevers at NIST after the MRR using field emission scanning electron microscopy showed tip wear and debris attachment to the end of the cantilever. This was likely due to the contact between the tip and surface during force curve measurements necessary for the reference cantilever method. The observation suggests that one potential issue in a wider round robin might be the effect of a changing tip morphology (tip wear) on calibration results. As more participants test the same cantilever, this cumulative damage effect may become more significant. It is also anticipated that sharper Si cantilevers may be more sensitive to this effect, therefore procedural limitations (e.g., limiting the amount of force or the stroke length actually applied during force curve testing) should be implemented to limit cumulative damage from a large round robin among many participants.

## 8. Conclusions and Recommendations for a Future Round Robin

There are several results from this study that reveal important information about the potential accuracy and precision of the spring constant measurement techniques used as well as suggestions for improving handling and reporting that could streamline future round robins in this field.

As far as improving the conducting of future round robins, there are several recommendations. They are summarized here in bulleted form as:

- Include flat bladed tweezers in test “kit” to reduce chip handling damage
- Break off unused cantilevers from test chips
- Use chip rocking/twisting method of removal from storage gel
- Report to three or more significant figures
- Electronic spreadsheet format with consistent calculation documentation should be used to reduce the possibility of transcription and calculation errors.

In addition, systematic characterization of the sample and reference cantilevers prior to and after testing may help document the effects of a large number of participants on the validity of round robin results on such methods where small scale changes may have considerable influence. Optical micrographs at several scales and resonance frequency measurements of the cantilevers are suggested as monitoring tools.

The number of test cantilevers (six) used in the MRR study was, in retrospect, excessive and increased the workload while offering little additional insight. It is suggested that the number of primary samples be reduced to one or two in future studies with the additional focus being put onto providing a wider range of cantilever types (material, shape, size, range of spring constants) that cover the needs of the community. While this MRR was conducted without loss of either test or reference cantilevers (at least the ones that counted), it is anticipated that a wider round robin with more participants would increase the likelihood of accidental damage to the samples and thought should therefore be given to providing “backup” specimens (both test cantilever and reference artifacts) “just in case” such that participants later in the study are given their chance to contribute to their full potential.

All of the spring constant calibration methods performed well for this type of cantilever which was a silicon nitride triangular cantilever with a spring constant of approximately 0.4 N/m. Not only did all the techniques have adequate precision (under ± 7 %), they all agreed within 11 % of an SI traceable benchmark value. The reference cantilever method has the potential for SI traceability and we are currently microfabricating a production batch of reference cantilever arrays [5] that would have their accuracy benchmark established through linkage to EFB measurements.

The thermal method seems very capable of providing precise spring constants with reasonable ease of use. In the case of the cantilevers tested, the AFM methods are accurate to within 10 %. An LDV thermal technique, currently under developmentat NIST, demonstrated a precision and accuracy similar to the SI traceable EFB technique for the cantilever tested. We are currently exploring the validity of these measurements over a wider range of cantilevers using LDV Thermal and EFB to see if it the observed accuracy continues to persist.

A larger round robin currently underway in VAMAS TWA29 will look at expanding the results of this MRR by providing a wider range of cantilevers (different size, shape, material, tip, and spring constant) in order to provide a fuller picture of the capabilities of these different calibration techniques among different laboratories around the world.

## Figures and Tables

**Table 1:** Comparison of precision and accuracy for different calibration techniques using Test Cantilever #3

Calibration Technique	Average k (N/m)	Type A Uncertainty† (± 1 sd, %)	Comparison to Benchmark‡ (%)
Added Mass	0.392	5.4	+4 %
Reference Cantilever	0.370	6.8	−2 %
LDV Thermal	0.3781	1.8	0 %
AFM Thermal (A)	0.410	4.4	+9 %
AFM Thermal (B)	0.381	3.9	+1 %
EFB	0.3764	1.6	0 %(benchmark)

† Also termed random uncertainty (obtained using statistical methods)

‡ Relative to EFB benchmark

**Table 2:** Comparison of precision and accuracy for different calibration techniques using Test Cantilever #5

Calibration Technique	Average k (N/m)	Type A Uncertainty† (± 1 sd, %)	Comparison to Benchmark‡ (%)
Added Mass	0.427	5.2	+11 %
Reference Cantilever	0.407	5.2	+6 %
LDV Thermal	0.3808	1.6	−1 %
AFM Thermal (A)	0.365	1.6	−5 %
AFM Thermal (B)	0.360	1.0	−6 %
EFB	0.3841	1.4	0 % (benchmark)

† Also termed random uncertainty (obtained using statistical methods)

‡ Relative to EFB benchmark

**Fig. A1:**
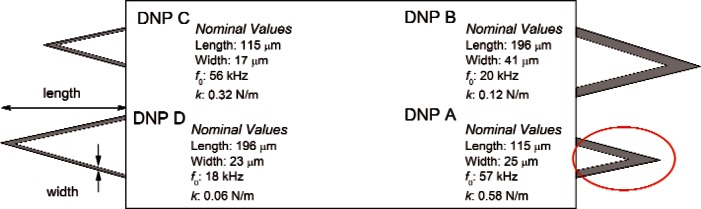
Schematic for test cantilever chips showing locations of cantilevers.

**Fig. A2:**
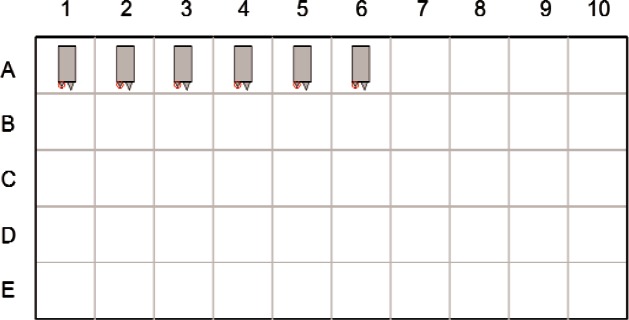
Orientation and placement of the test cantilever chips within the sample box.

**Fig. A3:**
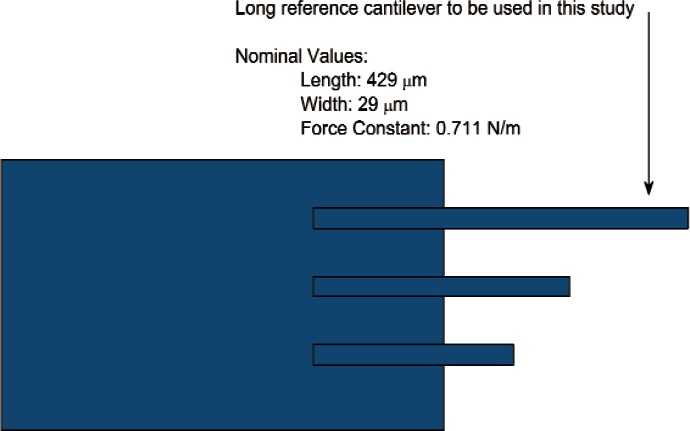
Reference cantilever chip used for this study.

**Fig. B1:**
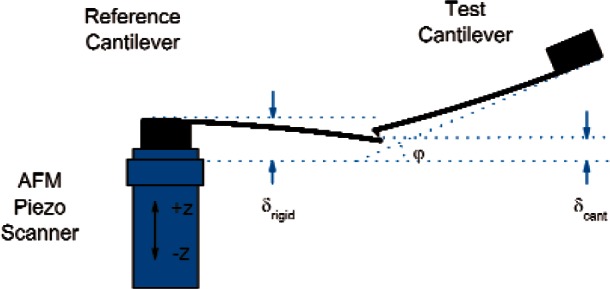
Schematic diagram of the test cantilever pressed against the reference cantilever.

**Fig. B2:**
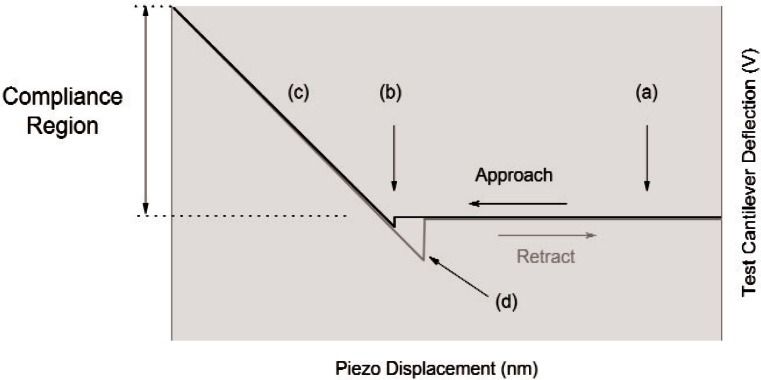
Force displacement curves of the approach and retract portions.

**Fig. B3:**
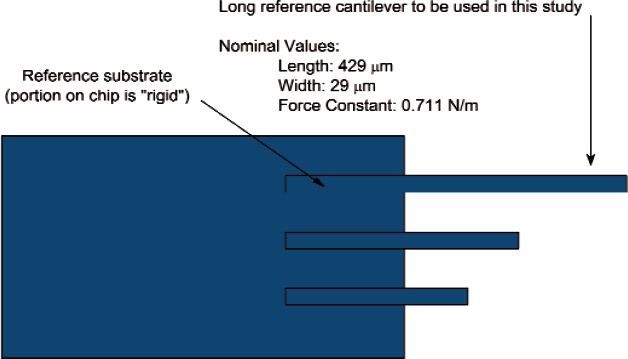
Reference cantilever chip.

**Fig. B4:**
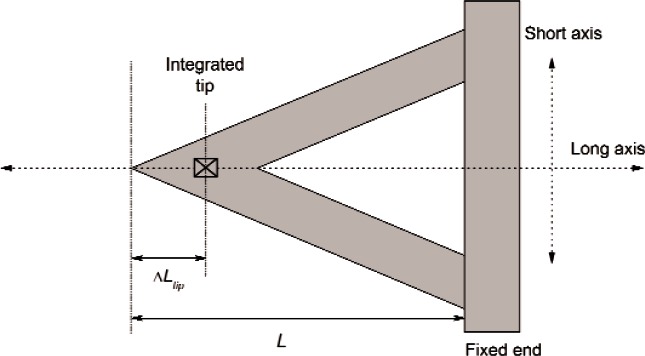
Triangular test cantilever diagram showing location of the integrated tip.

**Fig. C1:**
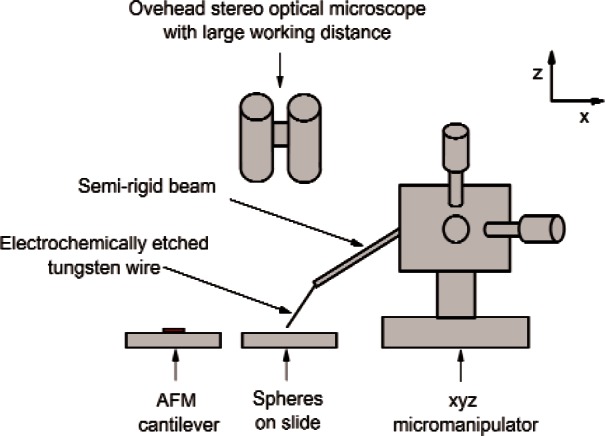
Schematic diagram of pick up and deposition of spheres.

**Fig. C2:**
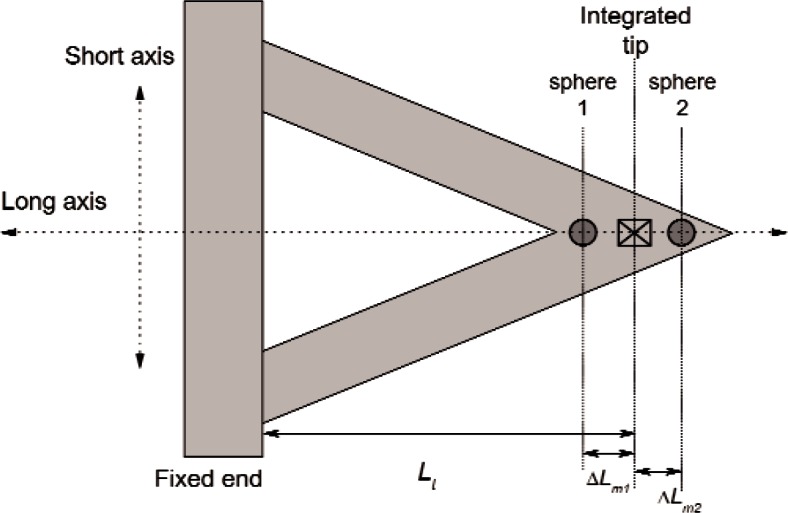
Placement of the sphere on the center line of the cantilever.

**Fig. C3:**
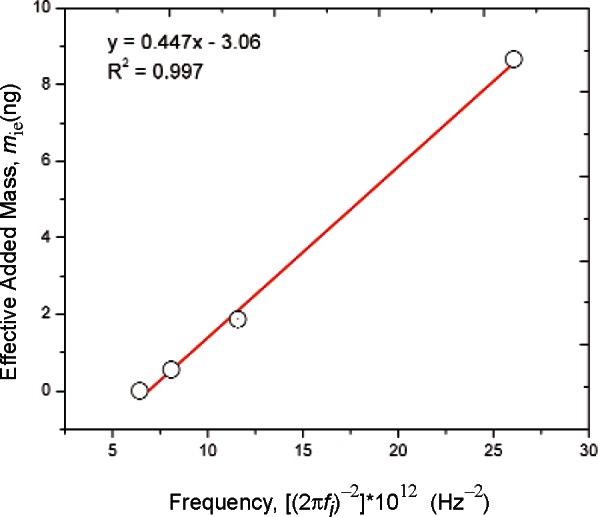
Added mass method spring constant calculation plot.

## Footnotes

1 Unless otherwise specified, uncertainties expressed in this paper are ± 1 standard deviation and relative uncertainties are ± 1 standard deviation / mean and expressed in %.

2 Certain commercial equipment, instruments, or materials are identified in this paper to adequately specify the experimental procedure. Such identification does not imply recommendation or endorsement by the National Institute of Standards and Technology, nor does it imply that the materials or equipment identified are necessarily the best available for the purpose.

## APPENDIX A. AFM Cantilever Spring Constant Calibration VAMAS Mini Round Robin Test Kit

The test kit consisted of six contact mode cantilever chips (DNP cantilever, Veeco Probes, Camarrillo, CA) and a reference cantilever chip (CLCF-NOBO, Veeco Probes, Camarrillo, CA) and were supplied by the vendor. The test cantilevers were taken from similar locations within a wafer production batch and were selected for anticipated consistency in spring constant. The cantilever to be tested was the “DNP A” cantilever on each chip. In the colloquial terminology of the chip where the cantilever legs are described in relative terms as either “fat” or “thin” and either “short” or “long,” the “DNP A” test cantilever fits the description of “short-fat.” The relative location of the DNP A cantilever on the chip is shown in Fig. A1. The nominal spring constant, provided by the manufacturer for this cantilever, is 0.58 N/m. The nominal tip height is 3 μm. The six chips were placed on a “X4 Gel-Pak” storage box (Gel-Pak, Hayward, CA) using the first six places in row “A” as shown below in Fig. A2. For reporting purposes, these test cantilevers were described as samples 1–6.

The storage box allowed safe shipping of the chips during the mini round robin. The chips were oriented so that the cantilever of interest (“A”) was located toward the lower left corner of each chip. The three unused cantilevers on each chip were not intentionally altered in any way.

The other part of the test kit consisted of the reference cantilever chip. To minimize potential damage to the reference cantilever from handling, it was mounted in the middle of a steel puck using double sticky “press tab” adhesive. The reference chip consisted of three reference cantilevers of different length (Fig. A3). For the purposes of this study, only the longest reference cantilever was used. The manufacturers nominal specifications for dimensions (length = 429 μm) and spring constant (0.711 N/m) were used for calculations requiring these parameters. The reference cantilever chip was placed into its own plastic box to protect it during shipping. A magnet glued to the bottom of the box using pressure sensitive adhesive allowed the steel puck to be secured magnetically within the box for shipping. It could be removed from the box and placed into the stage of the AFM for the calibration step without having to touch the actual silicon chip itself.

## APPENDIX B. AFM Cantilever Spring Constant Calibration Reference Cantilever Method

### 1. Scope

This method covers the calibration of the spring constant in the *z* (vertical) direction for Atomic Force Microscope (AFM) cantilevers using a reference cantilever.

### 2. Referenced Documents

#### 2.1

Torii, A., Sasaki, M., Hane, K. and Okuma, S. A method for determining the spring constant of cantilevers for Atomic Force Microscopy. Measure-ment Science and Technology. **7**, 179 (1996).

#### 2.2

Tortonese, M. and Kirk, M. Characterization of application specific probes for SPMs SPIE **3009**, 53 (1997).

#### 2.3

Gates, R. S., and Reitsma, M. G., Precise atomic force microscope cantilever spring constant calibration using a reference cantilever array. Rev. Sci. Instr. **78**, 086101 (2007).

### 3. Terminology

#### 3.1

*Test cantilever:* a cantilever to be calibrated.

#### 3.2

*Reference cantilever:* a cantilever of known spring constant (supplied for this method).

#### 3.3

*Reference substrate:* microfabricated chip containing the reference cantilevers (Figs. B1 and B3).

#### 3.4

*Compliance region of force-displacement curve:* the elastic deflection portion of an AFM cantilever when pressed against another material (Figure B2).

#### 3.5

*Intrinsic spring constant:* the stiffness of a cantilever perpendicular to its long axis.

#### 3.6

*Effective spring constant:* the stiffness of a cantilever perpendicular to the surface being probed.

### 4. Significance and Use

The reference cantilever method is used to calibrate AFM cantilever spring constants. This procedure is for the *z* (vertical) direction flexural spring constant and can be applied to rectangular and triangular cantilevers, whether coated or uncoated, with sharp tips or colloidal probes. The basic assumption for this procedure is that the spring constant of the reference cantilever used for calibration should be in the same order of magnitude with the test cantilever spring constant.

### 5. Summary of Test Method

The method utilizes a reference cantilever of known spring constant. The reference cantilever is placed on the sample holder which was then placed on the sample stage of an AFM. A test cantilever of unknown spring constant is placed in the AFM cantilever holder and aligned with the laser deflection-sensor optics just as it would be for normal imaging operation. The test cantilever is brought into close proximity to the reference cantilever and a series of measurements are made using the force-displacement curve mode of the AFM.

Figure B1 shows the AFM configuration for this method where the reference cantilever mounted on a (*z*) scanning piezo AFM sample stage (Veeco Multimode® AFM used in this study) [it will also work in configurations in which the upper test cantilever is mounted on a (*z*) scanning piezo holder]. The test cantilever is secured above the reference cantilever in a cantilever holder.

In order to perform the reference calibration method, the test cantilever *z* deflection must be measured on both the reference cantilever (*δ*_cant_) and a rigid surface (*δ*_rigid_) approximated by the reference substrate. Conceptually, this is shown below.

*δ*_rigid_: The test cantilever is placed into contact with the thick, rigid, portion of the (silicon) reference substrate, shown in Fig. B1. The deflection of the test cantilever on this surface is measured as the substrate is moved vertically by an amount *δ*_rigid_.
*δ*_cant_: The test cantilever is placed into contact with the flexible free end of the reference cantilever, as illustrated in Fig. B1, and the deflection of the cantilever under test, *δ*_cant_, is measured as the base of the reference cantilever moves vertically by the amount *δ*_rigid_.

The relationship between the spring constant of a test cantilever, the reference cantilever, and the deflections measured during contact were originally given by Torii et al., [2.1] for horizontal cantilevers and by Tortonese and Kirk [2.2] for an inclined test cantilever. Unfortunately, an error in the derivation in reference 2.2 placed the cos *φ* term in the denominator when it actually belongs in the numerator as a cos^2^ *φ* term [2.3]. For an 11° incline in the test cantilever this difference is about 6 %. The correct form is:

(B1) $$k_{test}=k_{ref}\left(\frac{\delta_{rigid}-\delta_{cant}}{\delta_{cant}}\right)\cos^{2}\varphi .$$

In practical terms, the cantilever calibration is accomplished by recording the force-displacement curves for both (rigid surface and the test cantilever) cases and measuring the slopes of the straight-line (compliance) portions of the data. The actual units used for the displacement curves do not matter as long as they are consistent since they will cancel out. Typically the test cantilever deflection is recorded as a voltage from the laser-position-sensitive photodetector and the piezo displacement is given in nm. A typical force-displacement curve is shown in Fig. B2.

In the “Approach” portion, the piezo (with attached reference cantilever/substrate) first moves upward in the +*z* direction (Fig. B1) toward the test cantilever. The test and reference are out of contact at (a) and no deflection in the test cantilever occurs. Contact between the test cantilever and reference cantilever occurs at (b), sometimes accompanied by a small “snap-on” as the surfaces are brought into such close proximity that surface attractions pull them together.

As the sample continues to translate in the +z direction the test cantilever continues to deflect at the same speed as the surface it is touching. The region along (c) is called the *compliance region*.

For ideal elastic materials free of interferences, the compliance region portion of the trace should be linear. For the “retract” portion of the force curve, the piezo scanning direction is reversed (− *z*; see Fig. B1), and the compliance region along (c) is traversed again. Often, attractive forces between the test cantilever tip and the surface cause the surfaces to stay together until point (d) when the tip “snaps off” the surface. From then on, the piezo completes its scan with the surfaces out of contact once again along (a).

Calculation of the test cantilever spring constant is performed according to the steps suggested in ref. [2.2].

*S*_rigid_ is the slope of the compliance region when the test cantilever is in contact with the reference substrate; typically, this value is given in volts per nanometer (V/nm) but volts per volt (V/V) is also commonly encountered.

*S*_cant_ is the slope of the compliance region when the test cantilever is in contact with the free end of the reference cantilever.

If the normal spring constant of the reference cantilever at the actual point of contact of the tip is k_ref_, then the normal spring constant of the test cantilever, k_test_, can be calculated as

(B2) $$\begin{array}{l} \;k_{\text{test}}=k_{\text{ref}}\left(\frac{S_{\text{rigid}}-S_{\text{cant}}}{S_{\text{cant}}}\right)\cos^{2}\varphi \\ \text{or}\\ \;k_{\text{test}}=k_{\text{ref}}\left(\frac{S_{\text{rigid}}}{S_{\text{cant}}}-1\right)\cos^{2}\varphi \end{array}$$

where *φ* is the angle between the Test cantilever and the horizontal (Fig. B1). This angle value is supplied by the AFM manufacturer.

Note that the spring constant (*k*_test_) calculated by this method is the “intrinsic” spring constant perpendicular to the long axis of the cantilever. AFM’s use a cantilever that is tilted with respect to the surface being probed; therefore, intrinsic spring constants must be divided by cos^2^ *φ* to yield the “effective” spring constant for the cantilever in that particular AFM.

### 6. Atomic Force Microscope Instrumentation

While the procedure can be used on any AFM, the procedure is written based on a topview optics system. The general requirement for the AFM are:

#### 6.1

The AFM must be equipped with an optical microscope capable of viewing a mounted reference cantilever and test cantilever simultaneously in order to align and superimpose them with a reasonable degree of accuracy. Tip placement accuracy should be 5 μm or better.

#### 6.2

AFM instrument must be able to acquire and save force-displacement curve data.

### 7. Materials and Preparation

#### 7.1 Optics

All optical instrumentation used for length/dimension measurement (including the AFM overhead optics) should be calibrated before use. We have found that one practical way to make rapid alignment measurements using video optics is to translate the cantilever a prescribed amount (e.g., cyclic scanning 10 μm) and note the extremes of motion on the video screen. A properly sized marker (e.g., 5 μm) applied to the video screen then provides a fiduciary comparison for estimating the tip location for aligning the test and reference cantilevers.

#### 7.2 Reference Cantilever

For this procedure, a commercial reference cantilever chip (Veeco CLFC-NOBO) has been supplied. The chip consists of three reference cantilevers. The reference cantilever to be used is the longest cantilever in the set as illustrated in Fig. B3.

#### 7.3

Measure the position of the Test cantilever tip, *L*_tip_ record an optical image of the integrated tip (cantilever inverted) and measure the distance between the integrated tip apex and the end of the cantilever as illustrated in Fig. B4. This distance, *L*_tip_, must be considered in order to accurately locate the contact point of the tip on the reference cantilever.

In practice, for a triangular test cantilever, this can be accomplished by visually estimating the relative position of the integrated tip with respect to the two “V” portions of the test cantilever. The integrated tip apex relative location will be in the same spot when the cantilever is flipped over as it would be for any top-view optical system.

### 8. Procedure

The following procedure was written specifically for an overhead optical AFM instrument (for example, Veeco Multimode). Variations of this procedure may be required for other experimental setups.

#### 8.1

Adjust the xy positioning of the AFM head to make sure it is roughly centered over the sample stage. Ensure the AFM sample stage is well lowered (i.e., “tip up”), and then insert the test cantilever (in holder) into the AFM head and clamp it in place.

#### 8.2

Focus the overhead optics onto the test cantilever. It will be pointing to the left as shown in figure B4. Once a clear image of the test cantilever can be seen, adjust the optics xy position to place the cantilever on the right side of the field of view. From this point on, do not adjust the xy positioning for the overhead optics. Remove the test cantilever holder from the AFM head or move the tip away from the surface sufficiently to allow insertion of the reference cantilever puck.

#### 8.3

Place the reference cantilever sample puck onto the center of the AFM sample stage without adjusting the overhead optics in x or y. Focus the overhead optics onto the reference cantilever chip. Carefully move and rotate the reference cantilever puck using tweezers so that the reference cantilever to be used is in the field of view and pointing horizontally and to the right on the viewing screen as shown in figure B3.

#### 8.4

Place the test cantilever (in holder) into the AFM head and clamp it into place.

#### 8.5

Using the xy positioning for the AFM head, ensure that the position of the test cantilever can be adjusted such that it can reach both the free end of the reference cantilever to be used, as well as the reference substrate.

#### 8.6

Position and optimize the AFM laser optics on the test cantilever.

#### 8.7

Lower the AFM head (“tip down”) until the test cantilever is close to the reference cantilever. This can be done by focusing the overhead optics on the reference cantilever and lowering the AFM head in small increments until the test cantilever comes into view, but not quite into focus.

#### 8.8

Using the xy positioning of the AFM head, move the test cantilever over to the free end of the reference cantilever to be used. Align the centerline of the long axis of the test cantilever with that of the reference cantilever (Fig. B4). Align both cantilevers such that the end of the test cantilever coincides with the end of the reference cantilever. Using the end of the reference cantilever as the zero point, adjust the AFM head position so that the end of the test cantilever is positioned at 2×*ΔL*_tip_ (see 7.3) from the end of the reference cantilever. This will place the tip of the cantilever at a contact point *ΔL*_tip_ from the end of the reference cantilever.

#### 8.9

As a start, use a force curve ramp size of 500 nm and engage the samples as described in the AFM instrument manufacturer’s instruction manual. Once the tip has engaged the sample, a force curve can be acquired and saved. Ensure that the constant compliance region (Fig. B2) of the acquired force-separation curve is linear. If bowing is seen, reduce the *z* scan size and acquire another force-separation curve. Note that both *δ*_rigid_ and *δ*_cant_ should be recorded at the same ramp size. Save the force curve data under an appropriate name (e.g., rigid01).

#### 8.10

Before adjusting the xy positioning of the AFM head, make sure the reference and test specimens are well separated in the *z* direction (i.e. tip retracted). Position the test cantilever over a clean area of the substrate base of the reference cantilever (Fig. B3).

#### 8.11

Lower the AFM head until the test cantilever is a few microns above the reference substrate. This can be done by focusing the overhead optics onto the reference substrate surface and lowering the AFM head in small increments until the Test cantilever comes into view, but not quite into focus.

#### 8.12

Engage the sample. Acquire and save the force curve data as before. The two force curves (on reference cantilever and substrate) constitute the data pair that is used in calculating the spring constant.

#### 8.13

Repeat 8.6–8.12 to acquire further force curve measurements. At least six (6) measurement pairs should be recorded. Each data set pair should be adequately labeled to reveal the pairing (e.g. Cant01, Rigid01 etc.) to facilitate later data analysis. Minimal delay (<3 minutes) should be allowed between the two measurements in a pair of measurements to minimize instrument drift effects.

### 9. Sources of Error

The largest potential source of error lies in the location of the tip on the reference cantilever. Since for a rectangular cantilever beam the spring constant changes with the length *cubed*, even small errors can significantly affect the final spring constant estimate.

Another source of error lies with the force curves themselves. Since these curves represent actual contact between a tip and a surface, it is subject to many real-world effects such as friction and adhesion. This is compounded by the fact that two curves are required for a single calibration so that the errors are cumulative.

### 10. Precautions

Care should be taken to ensure that the z scan range in the force curve does not exceed the linear range of the photodetector / optical lever system. Excessive force applied between the test cantilever and a surface may also cause damage to the tip and buckling of the end of the cantilever.

### 11. Results Reporting and Adjustment

For each force-separation curve, isolate the compliance region portion of the data (shown in Fig. B2) and determine the slope of this region. It is recommended that a graphical assessment of the analyzed portion of the data also be made to ensure no artifacts are included in the data analysis.

Indicate how the compliance slope is determined (e.g., linear regression fit to ASCII data; using a software package) and how much of the compliance curve data is used.

#### 11.1 Results Reporting Table

Table B1 is an example of how the raw data should be recorded (electronic spreadsheet format is preferable).

**Table B1:** Raw data reporting format for reference cantilever method

Cantilever ID	Δ*L*_tip_ μm	*φ*	Cos^2^ *φ*	Ramp size nm	Test #	*S*_rigid_ V / nm	*S*_cant_ V / nm
DNP 1A	5.0	11°	0.964	300		Approach or Retract	Approach Retract
					1	0.0234	0.0145
					2	0.0221	0.0151
					3	0.0229	0.0157
					4	0.0235	0.0142
					5	0.0226	0.0146
					6	0.0238	0.0153

#### 11.2 Sample Calculation

The nominal value given for the spring constant of the reference cantilever is the estimated stiffness at the end of the beam. In this method, however, the load is applied to the reference cantilever at a distance of Δ*L*_tip_ from the end (see 7.3) which will result in a slightly greater stiffness. To correct the calculated data for the distance between the point of contact between the two cantilevers (Δ*L*_tip_) and the end of the reference cantilever we need to apply an *off-end loading correction.* Since *k* (spring constant) varies as the cube of the length (*L*), the off-end correction is applied as:

(B3) $$k_{\text{ref}}=k_{\text{end}}{\left(\frac{L}{L-\nabla L_{\text{tip}}}\right)}^{3}$$

where *L* is the length of the reference cantilever from its fixed end to its free end and *k*_end_ is the spring constant of the reference cantilever defined at the end of the cantilever. For the reference cantilever used in this work, the nominal value provided by the supplier is *k*_end_ = 0.711 N/m.

The value of *k*_ref_ is then used in Eq. (B2). Using the value of *φ* for this example AFM (11°) in Eq. (B2), along with the other data shown in Table B1, *k*_test_ is calculated for each measurement as shown in Table B2. The average, standard deviation and relative standard deviation (RSD) of the six measurements should also be provided in the table. If data is entered into an electronic spreadsheet then Table B2 could actually be an extension of Table B1 and the proper formulas could be entered in each calculation cell. This is the preferred format.

**Table B2:** Calculated data reporting format for reference cantilever method

Test #	*L* μm	Δ*L*tip μm	*k*_ref_ N/m	*k*_test_ N/m
	429	5.0	0.736	
1				0.436
2				0.329
3				0.326
4				0.465
5				0.389
6				0.394
			Avg	0.390
			Std Dev.	0.056
			RSD, %	14.3

## APPENDIX C. AFM Cantilever Spring Constant Calibration Added Mass Procedure

### 1. Scope

This procedure covers the calibration of Atomic Force Microscope (AFM) cantilever spring constants in the *z* direction (vertical) using the added mass (“Cleveland”) method, modified for off-end corrections.

### 2. Referenced Documents

#### 2.1

Cleveland, J. P., Manne, S., Bocek, D., Hansma, P. K. A nondestructive method for determining the spring constant of cantilevers for scanning force microscopy. Review of Scientific Instruments. 64 (2), 403 (1993).

#### 2.2

Sader, J. E., Mulvaney, P., and White, L. R. Method for the Calibration of Atomic Force Microscope Cantilevers. Review of Scientific Instruments 66 (7), 3789 (1995).

#### 2.3

Sader, J. E. Parallel beam approximation for V-shaped atomic force microscope cantilevers. Review of Scientific Instruments 66 (9), 4583 (1995).

### 3. Terminology

#### 3.1

*AFM*: Atomic Force Microscope

#### 3.2

*Resonance frequency f*: is the first bending mode resonance frequency, perpendicular to the long axis of the cantilever.

#### 3.3

*Cantilever resonance frequency*: *f*_0_ is the resonance frequency of the cantilever without added mass.

#### 3.4

*Test cantilever*: cantilever to be calibrated.

#### 3.5

*Cantilever holder*: AFM cantilever holder, which is used to mount the test cantilever in AFM.

#### 3.6

*Loaded resonance frequency*: *f*_i_ is the resonance frequency of the cantilever measured with an added mass (*m*_i_).

#### 3.7

*Cantilever tip*: the actual tip apex (point of contact) that is made with the surface when an AFM cantilever is used. The length of the cantilever from the fixed base to the tip is designated as *L*_t_.

#### 3.8

*Cantilever end*: the free end of the cantilever. The length of the cantilever from the fixed base to the free end is designated as *L*_e_.

#### 3.9

*Intrinsic spring constant*: the stiffness of a cantilever perpendicular to its long axis.

#### 3.10

*Effective spring constant*: the stiffness of a cantilever perpendicular to the surface being probed.

### 4. Significance and Use

The added mass method is used to calibrate the intrinsic spring constant of AFM cantilevers. It can be applied to rectangular, triangular, coated or uncoated cantilevers with sharp tips or colloidal probes. The key requirements are that the locations and the masses of the spheres added for frequency measurements can be measured accurately.

### 5. Summary of Test Method

#### 5.1

The first flexural mode resonance frequency, *f*_0_, of the test cantilever is measured.

#### 5.2

A tungsten or gold sphere is placed at the free end of the cantilever.

#### 5.3

The size and position of the sphere on the cantilever is measured (e.g., using a suitable calibrated microscope).

#### 5.4

The resonance frequency of the cantilever with attached sphere is measured. The sphere is then removed.

#### 5.5

Steps 5.2—5.4 are repeated for at least 2 spheres (3 point plot). A larger number of data points (e.g., 5 spheres (6 point plot)) are desirable to reduce the statistical uncertainty. The range of sphere size depends on the spring constant but in general 5 μm to 15 μm size spheres are used.

#### 5.6

The mass of each spherical mass added (*m_i_*) is calculated from the measured diameter and known density of the material.

#### 5.7

The general relationship between added mass, *m_i_*, and resonant frequency, *f_i_*, is

(C1) $$m_{i}=k{\left(\frac{1}{2\pi f_{i}}\right)}^{2}-m^{*}$$

where *k* is the spring constant of the cantilever. The quantity m^*^ is called the “effective mass” of the cantilever. If several known masses are added to the end of a cantilever and resonance frequencies are measured for each added mass, a linear plot of added mass *m_i_* versus (2π*f*_i_)^−2^ will give a straight line with slope of *k* and an ordinate intercept of − m^*^.

### 6. Atomic Force Microscope Instrumentation

This method requires an AFM instrument with hardware and software suitable for cantilever resonance frequency measurement.

### 7. Materials and Preparation

This method uses a sharp tungsten wire to pick up, maneuver and attach spherical particles to the cantilever. It relies on attractive meniscus forces to pick up the spheres; therefore it may be sensitive to changes in relative humidity. The relative humidity of the laboratory used for this work was 45 % ± 5 %.

#### 7.1 Spheres

Powder consisting of spherical gold or tungsten spheres. For this purpose, 325 mesh spherical gold powder can be used (Alpha Aesar #43900, 99.9 % purity on a metals basis). Tungsten spherical particles donated by Asylum Research (Santa Barbara, CA) are also available to participants.

#### 7.2 Tungsten wire (for suggested sphere mounting apparatus in 7.5)

For this work, 0.25 mm diameter (Alpha Aesar #10408, 99.95 % purity) tungsten wire was used to create a sharp micromanipulator probe tip to manipulate the spherical particles. Other wire materials that can be sharpened to a fine point may also be suitable.

#### 7.3 Electrochemical etching of a tungsten wire

There are a number of different techniques available for tungsten wire etching. It is left to the user to decide the best method by which to electrochemically etch the end of a tungsten wire down to a fine point (ca. 100 nm radius of curvature). Note that too fine a point is not desirable since one wants a large enough area of contact for the sphere to adhere to the tip when the meniscus forms between the contacting surfaces.

#### 7.4 Optical Microscope

All optical instrumentation used for length / dimension measurement must be calibrated before use.

#### 7.5 Suggested sphere mounting apparatus

Figure C1 depicts the sphere mounting apparatus used at NIST. Participants can use other setups but details on the apparatus and procedure used should be provided. The following are the major components of the apparatus used.

##### 7.5.1 Optical microscope

A stereo microscope with a long working distance was used to allow simultaneous observation and micromanipulation. A field of view of 500 μm or less is necessary to allow location, pickup, and placement of small spheres onto the cantilevers with sufficient control.

##### 7.5.2 Sphere slide

Tungsten or gold spheres are placed onto a clean glass microscope slide in such a way as to provide a large number of isolated spheres that can be picked up with the tungsten probe.

##### 7.5.3 Probe translation stage

An xyz translation stage capable of at least 10 mm travel in each direction is used to manipulate the tungsten probe above the surfaces. Micrometers on each axis provide translation adjustment of each axis.

##### 7.5.4 Etched tungsten wire

An electrochemically etched tungsten wire attached to a semi-rigid rod was used to maneuver the spheres (Fig. C1). An example of such a rod would be a tool steel rod approximately 3 mm in diameter and 150 mm long.

##### 7.5.5

Pickup and deposition of the spheres is best accomplished with a combination of orthogonal (xyz) mechanical translation axes and haptic (tactile feedback) controls as shown in Fig. C1. The translation stage is used for coarse adjustment of the probe to a location just above the surface. By exerting gentle pressure on the semi-rigid beam with one’s fingers the operator can cause the tungsten probe tip to smoothly approach the surface in the proximity of a sphere (ca. 10 μm to 20 μm travel). Release of pressure then causes the tip to retract smoothly from the surface.

### 8. Procedure

Using “non-critical” cantilevers for practice, it is recommended that the individual user decide the micromanipulation method most suitable to them for mounting and removing spheres. It is also recommended that each user be well rehearsed in their chosen technique before proceeding to calibrate the VAMAS cantilevers. The following steps are based on the suggested sphere mounting apparatus described in 7.5 above.

#### 8.1

Ensure the AFM head is raised with sufficient clearance above the sample and place the cantilever holder (containing the test cantilever) into the AFM head. Lock it into place. Focus and optimize the AFM laser optics onto the test cantilever and perform the resonance frequency analysis on the cantilever. Sometimes this is referred to as “tuning” the cantilever. Once the resonance frequency for the test cantilever has been recorded, remove the holder from the AFM instrument and transport it to the sphere mounting apparatus under the stereomicroscope (Fig. C1).

#### 8.2

Ensure the surface of the sphere slide is slightly higher (*z* axis) than the cantilever in the cantilever holder (Fig. C1). Focus the overhead optics onto the sphere slide and select a uniform, symmetric sphere.

#### 8.3

Lower the tungsten tip to within several micrometers of the target sphere and use gentle, fine motion to establish contact between the tip and sphere. If performing fine motion by hand, a small force is applied to the semi-rigid beam to traverse the final distance and establish contact with the sphere. By controlling the (finger) pressure to the beam, spheres can be contacted and picked up in a single, smooth, down-up motion. Furthermore, since spheres can move around slightly on the slide before they stick to the tungsten tip, it is found that finger control offers more freedom of movement and can thus be a more effective technique for pick up than using the micromanipulator adjustment micrometers alone.

#### 8.4

After the target sphere has been picked up, make sure the tungsten tip is raised enough before removing the sphere slide and replacing it with the cantilever holder. Focus the overhead optics such that both the tip and the cantilever below it can be seen.

#### 8.5

Focus the overhead optics onto the cantilever and then carefully lower the tungsten tip down to within several micrometers of the cantilever. Using the same technique as described in 8.3, place the sphere on the end of the cantilever (avoid contact with the integrated tip of the cantilever). Place the sphere close to the centerline of the long axis of the cantilever near the integrated tip (Fig. C2).

#### 8.6

Once the sphere has been placed onto the cantilever, raise the tungsten tip clear. Record an image of the sphere on the cantilever. Ensure that two measurements can be made from the image(s): (1) The diameter of the sphere, and (2) the position of the (center of) sphere relative to the integrated tip.

#### 8.7

Place the holder (containing the test cantilever) into the AFM head and lock it into place. Focus and optimize the AFM laser optics (on the test cantilever) and perform a resonance frequency analysis on the cantilever as described in 8.1. Once the resonance frequency for the test cantilever has been recorded, remove the holder from the AFM instrument and transport it to the sphere mounting apparatus. At this point it is wise to record an additional image of the sphere location on the cantilever to make sure it has not moved during the resonance frequency measurement.

#### 8.8

In the same fashion as described in 8.3, remove the sphere from the cantilever.

**CAUTION:** This is often the most difficult and potentially damaging part of the procedure. If contacting the sphere with the tungsten tip proves unsuccessful, there are several alternatives that can be tried. Switching to a less sharp tungsten tip (stronger meniscus forces) that can more easily pick up the sphere usually helps but the sharper tip must be switched back for the next sphere placement. Spheres can often be removed by very carefully “flicking” (oscillating) the end of the cantilever with the tungsten tip. **IMPORTANT: extra care is required to avoid catching the edge of the cantilever with the tungsten probe if this later technique is needed.** Alternatively, spheres can often be detached by driving the resonance externally with a moderately high amplitude (for example in an AFM). The sphere has been detached when the resonance peak jumps back to the initial (higher) resonance frequency determined in step 8.2 above.

Care must be taken to ensure that the sphere has actually been removed and has not merely moved to the underside of the cantilever. A resonance frequency measurement can confirm this (the frequency should return to the original resonance frequency (*f*_0_)).

#### 8.9

Repeat steps 8.2 – 8.8. It is recommended that a minimum of two sphere measurements are recorded, with a difference of >20 % in diameter for each new sphere added. This will be combined with the unloaded (no added mass) resonance frequency measurement to yield a three point plot. A five sphere (six total data point) plot is more desirable.

#### 8.10

Measure the unloaded resonance frequency of the test cantilever once again as a final step (the value should be within 0.5 % of that acquired before calibration).

### 9. Sources of Error

The largest potential for error lies in the sizing of the spheres and the estimates for the sphere placement on the cantilever. For this reason, a calibrated optical microscope with digital image capture capabilities is desirable.

### 10. Precautions

Care needs to be taken in placing the test cantilevers into the AFM holder to avoid breakage. Placement of the sphere onto the cantilever and removal of the spheres is potentially damaging to the cantilever and therefore requires caution.

### 11. Results Reporting

#### 11.1 Sphere Positioning and Off-Tip Correction

The spring constant of the cantilever should be determined at the integrated tip position. Since spheres should be placed along the long axis and they cannot be placed in the same position as the tip, it is important to note the position of the sphere relative to the integrated tip of the cantilever. In principle, measuring the resonance frequency of a test cantilever with a mass added on the free end means that the added mass probes the spring constant of the cantilever from its fixed end to the position of the mass. In order to make this correction later on, the position of the sphere relative to the integrated tip needs to be recorded. That is, the distance from the integrated tip apex to the center of the sphere.

Record the sphere positions according to the convention shown in Fig. C2. These offsets are then used to correct the added mass using equation C2 to provide the effective masses added (*m*_ie_) for sphere “i”. That is, spheres placed between the integrated tip and the free end of the cantilever (e.g., sphere 2) are negative values (− Δ*L*_m_) and will have the effect of increasing the effective added mass relative to the tip apex. Spheres placed between the tip and the fixed end (e.g., sphere 1) are positive values (+ Δ*L*_m_) and will have the effect of decreasing the effective added mass.

(C2) $$m_{\text{ie}}=m_{i}{\left(\frac{L_{t}-\Delta L}{L_{t}}\right)}^{3}.$$

Note that for this procedure, we are not taking off-axis loading into account (i.e., sphere placement away from the tip along the short axis). More information about the correction technique to be performed can be found in reference 2.2.

#### 11.2 Results Reporting Table

Below is an example of how the results should be recorded. Electronic (spreadsheet) format is preferable.

#### 11.3 Sample Calculation

The following is a sample calculation for the results shown in Table C1 above. The calculation includes the so-called Sader off-end correction (ref. 2.2), which corrects for sphere placements at some distance, Δ*L*_m_, away from the desired position of spring constant determination. For this method we want to determine the spring constant at the position of the integrated tip, since this is the point along an AFM cantilever at which loading normally takes place. For each single added mass, use *ρ* = 19300 kg·m^−3^ (density of gold), and *ρ* (4/3)π*r*^3^ to determine the mass, *m*_i_, of each sphere added. If tungsten is used, the density should be 19250 kg·m^−3^. Then apply the off-end correction Eq. (C2) to give the effective mass (*m*_ie_). The general relationship between added mass (kg), resonance frequency (Hz) and spring constant (N/m) Eq. C1) becomes more specific when *k* and *m* are defined for the actual location of the integrated tip:

(C3) $$m_{\text{ie}}=k{\left(\frac{1}{2\pi f_{i}}\right)}^{2}-m^{*}.$$

**Table C1:** Raw data reporting format for added mass method

Cantilever ID	*L* μm	-Sphere -material	Sphere number	Sphere diameter, μm	Resonance Frequency -kHz	ΔLm μm
DNP 6A	108	Gold	–	0	62.8	–
		19300	1	3.8	56.0	+1.9
		kg·m^−3^	2	5.7	46.8	−1.4
			3	9.5	31.2	−1.1
			–	0	62.8	–

A plot of the effective added masses (*m*_ie_) versus measured resonance frequency (2π*f*_i_)^−2^ should yield a straight line of slope *k* (the spring constant at the location of the integrated tip) as shown in Fig. C3.

Regression analysis should be used to calculate the slope, intercept and uncertainty in the slope estimation (standard error of slope estimate). All of these values can be reported on an extended version of table C1.

As the test measurement is the resonance frequency of the cantilever, there is no directionality to the measurement. As a result, the plot provides the “intrinsic” spring constant of the test cantilever directly. AFM’s use a cantilever that is tilted with respect to the surface being probed; therefore, when being used, intrinsic spring constants must be divided by cos^2^ *φ* (where *φ* is the tilt angle of the cantilever with respect to the horizontal surface of a sample) to yield the “effective” spring constant for the cantilever in that particular AFM.
